# A cross-sectional study assessing the association between online ratings and clinical quality of care measures for US hospitals: results from an observational study

**DOI:** 10.1186/s12913-018-2886-3

**Published:** 2018-02-05

**Authors:** Martin Emmert, Nina Meszmer, Mark Schlesinger

**Affiliations:** 1 2014-15 Harkness & Robert Bosch Fellow in Healthcare Policy and Practice; Department of Health Policy and Management, Yale University, School of Public Health, 47 College Street, New Haven, CT 06520 USA; 2 Friedrich-Alexander-University Erlangen-Nuremberg, School of Business and Economics, Institute of Management (IFM), Lange Gasse 20, 90403 Nuremberg, Germany; 3 Chair of Health Care Management, Lange Gasse 20, 90403 Nuremberg, Germany; 4 Yale University, School of Public Health, Room 304 LEPH, 60 College Street, New Haven, CT 06520 USA

**Keywords:** Online ratings, Public reporting, Patient satisfaction, Patient narratives, Quality of care

## Abstract

**Background:**

Little is known about the usefulness of online ratings when searching for a hospital. We therefore assess the association between quantitative and qualitative online ratings for US hospitals and clinical quality of care measures.

**Methods:**

First, we collected a stratified random sample of 1000 quantitative and qualitative online ratings for hospitals from the website RateMDs. We used an integrated iterative approach to develop a categorization scheme to capture both the topics and sentiment in the narrative comments. Next, we matched the online ratings with hospital-level quality measures published by the Centers for Medicare and Medicaid Services. Regarding nominally scaled measures, we checked for differences in the distribution among the online rating categories. For metrically scaled measures, we applied the Spearman rank coefficient of correlation.

**Results:**

Thirteen of the twenty-nine quality of care measures were significantly associated with the quantitative online ratings (Spearman *p* = ±0.143, *p* < 0.05 for all). Thereof, eight associations indicated better clinical outcomes for better online ratings. Seven of the twenty-nine clinical measures were significantly associated with the sentiment of patient narratives (*p* = ±0.114, *p* < 0.05 for all), whereof four associations indicated worse clinical outcomes in more favorable narrative comments.

**Conclusions:**

There seems to be some association between quantitative online ratings and clinical performance measures. However, the relatively weak strength and inconsistency of the direction of the association as well as the lack of association with several other clinical measures may not enable the drawing of strong conclusions. Narrative comments also seem to have limited potential to reflect the clinical quality of care in its current form. Thus, online ratings are of limited usefulness in guiding patients towards high-performing hospitals from a clinical point of view. Nevertheless, patients might prefer different aspects of care when choosing a hospital.

**Electronic supplementary material:**

The online version of this article (10.1186/s12913-018-2886-3) contains supplementary material, which is available to authorized users.

## Background

Online rating websites have become a popular tool for increasing transparency regarding the quality of care of health care providers [1–3]. Besides a scaled survey, several rating websites (e.g., Yelp, FindTheBest - HealthGrove, RateMDs) have implemented a narrative commentary field [4] so that patients can report on their experience in their own words. So far, literature has shown the increasing popularity of such websites when it comes to the number of ratings [2, 5, 6], the traffic rank [5, 7], and the awareness of the population [8]. What we further know is that a large proportion of quantitative online ratings [2, 5, 9–13] and patients´ narratives are positive [1, 3, 14].

However, literature has also raised concerns regarding the usage of online rating websites. First, the derived ratings are not risk adjusted and thus do not seem to be appropriate to represent a provider’s quality of care [15]. In addition, the presented results are vulnerable to fraud since ratings are totally or partly anonymous on some rating websites [16] (However, it also has to be mentioned that certain rating websites have implemented different measures to deal with the problems of anonymous ratings; e.g., the Dutch patient rating website Zorgkaart or the German rating website jameda). It is also important to mention that people providing feedback on health care via social media are presumably not always representative of the patient population, which might limit the usefulness for certain patient groups [16, 17]. Studies have further shown that national hospital rating systems in the US may generate confusion rather than clarity as they share few common scores [18, 19]. Finally, since the ratings are often based on only a few reviews and are mostly positive [5, 6, 11], the usefulness of the ratings for patients might be limited. (However, recent research from the Netherlands has demonstrated that information from social media which integrates the patient’s perspective can be important for health care inspectorates, especially for its enforcement by risk-based supervision of elderly care [20].) In this context, one study recently showed that the overrepresentation of positive comments in online reviews might enable ineffective treatments to maintain a good reputation [21]. Applying this finding to the health care provider rating context, it means that low-performing providers may be likely to have positive ratings, which could lead to sub-optimal provider choices.

It thus remains questionable whether patients should rely on online ratings when choosing a provider [15]. In cases where the ratings are strongly correlated with clinical quality of care measures, it might be easier for patients to single out the best performers, which would increase the usefulness of the ratings. In contrast, if the ratings are uncorrelated or even negatively correlated with clinical performance measures, the choice becomes harder since consumers must strike trade-offs among attractive attributes [22]. To date, there is little evidence regarding the association between online ratings and clinical performance measures for hospitals in the US. To the best of our knowledge, there is only one study available evaluating the association between quantitative online ratings and hospital performance metrics [23]. (Two similar studies are available but focus on the association between online ratings and performance metrics based on the individual provider level [24, 25]). Furthermore, no study refers to the association between narrative comments and clinical performance measures. In this context, the present study aims at adding further knowledge on whether both quantitative and qualitative patient satisfaction results displayed on US hospital rating websites demonstrate an association with clinical performance measures.

## Methods

This study was designed as a cross-sectional study by analyzing the association between online derived patient ratings and clinical measures for US hospitals. Thereof, we collected a random sample of 1000 online ratings for US hospitals from RateMDs (04/2015). The website RateMDs uses a five-point scaled rating system of star rating scores and narrative comments to ask patients about their overall impressions about hospitals. Consequently, the collected data contains quantitative ratings and narrative comments. Since the aim of this study was to assess the differences between the five rating scores, we stratified the sample by rating score and collected 200 ratings of each rating score. As a maximum, we collected a total of 20 ratings for each state with an equally distribution of rating scores. Thereof, we collected the first ten ratings of each state starting with the best hospital and the remaining ten ratings were collected by starting with the worst hospital. In case not enough ratings were available within one state, we filled up the missing data with hospital ratings from other states.

In a next step, we used qualitative content analysis to determine the topics discussed in the narrative comments [26, 27] by using previous evidence [28]. We therefore conducted a search procedure in Medline (via PubMed) to identify previously published categorization schemes for narrative comments related to hospital ratings (10/2014; not presented here in detail). The identified schemes served as a starting point and were further extended in an iterative process. Our developed categorization framework aimed to capture both the topics mentioned within the narrative comments and the sentiment. We therefore applied both deductive and inductive steps—i.e. new categories were added until a saturation of topics had been reached [28]. The final framework was applied during a pre-test of 100 randomly selected narrative comments. Next, the content of each narrative comment was classified according to our final framework with respect to both the topic and the sentiment as positive, neutral, or negative [29]. Two of the authors independently carried out the assessment. The inter-rater agreement between the two raters was assessed using Cohen’s kappa coefficient (weighted) and was calculated to be 0.813; 95 CI: 0.796–0.834). We then derived the overall sentiment of each comment as positive, negative, or neutral [30, 31], based on the proportion of positive topics in each comment.

The clinical quality measures were derived from the Hospital Compare database published by the Centers for Medicare and Medicaid Services (CMS) [32] and downloaded from Data.Medicare.gov. For our study purpose, we focused on non-disease specific clinical quality measures, since we expected the narrative comments to be non-disease related in most cases. In total, we included 29 quality measures related to healthcare associated infections (*N* = 4), readmissions, complications and deaths (*N* = 5), as well as timely and effective care (*N* = 20). We then assigned the hospital ID included in the CMS dataset to the hospitals in our RateMDs database and matched the two datasets before conducting our analysis.

All statistical analyses were carried out by means of SPSS V22.0 (IBM Corp, Armonk, NY, USA). Descriptive analysis included calculating the mean and standard deviation (SD) for the characteristics of narrative comments as well as rated hospitals. The Kruskal Wallis test was used to determine the differences in non-parametric data between the rating performance groups. Two approaches were used to learn more about the association between the online ratings and clinical measures according to the display on Hospital Compare. First, regarding nominally scaled measures, we checked for differences in the distribution across the scaled survey rating and sentiment categories by using the chi-square test. Second, regarding metrically scaled measures, we applied the Spearman rank coefficient of correlation to measure the association between online ratings and quality of care information; (none of our dependent variables was normally distributed according to the Shapiro-Wilk test; *p* < 0.001, data not shown here). The association was calculated by adjusting for hospital type, hospital ownership, and emergency service. We also analyzed the correlation between the lengths of comments and the evaluation results and between the overall patient experience derived from the scaled survey results and the narrative comments. Inter-rater agreement between the two raters was assessed using Cohen’s kappa coefficient (weighted). Observed differences were considered statistically significant if *p* < .05 and highly significant if *p* < .001.

## Results

### Systematic search procedure and categorization framework

Our search procedure identified one study which analyzes and categorizes narrative comments derived from online hospital report cards about the hospital experience from the UK and was taken as the initial basis for our categorization framework [33]. Further studies which deal with a slightly different question were also screened to capture comment categories [29–31, 34–36]. Our applied categorization scheme distinguishes between ten main categories: the received care, facilities, wait time, clinicians and staff, communication, costs of care, personal issues, acknowledgements, recommendations for or against using the hospital, and others. (See Additional file 1 for an overview of our final categorization scheme/codebook, a further description of the categories, and examples of positive and negative comments.)

### Content and sentiment analysis of the narrative comments

Table 1 provides an overview of the hospitals (*N* = 623) related to the 1000 analyzed online ratings. Most ratings relate to acute care (98%) and voluntary non-profit hospitals, and those who provide emergency services (97%).

**Table 1:** Characteristics of all hospitals in our sample compared with all US hospitals

Criteria	Characteristics	Sample Hospitals (*N* = 623; in %)	US Hospitals^a^ (*N* = 4861; in %)
Hospital Type	Critical Access Hospitals	2.0	25.8
	Acute Care Hospitals	98.0	73.7
	Childrens	0.0	0.5
Hospital Ownership	Government Hospitals	14.6	25.5
	Hospitals owned by physicians	0.9	1.1
	Proprietary Hospitals	17.8	16.2
	Voluntary non-profit Hospitals	66.7	57.2
	Tribal	0.0	0.0
Emergency Service	Yes	96.8	92.1
	No	3.2	7.9
Ability to receive lab results electronically	Yes	81.1	67.4
	No	14.3	20.5
	Not Available	4.6	12.1
Ability to track patients’ lab results, tests, and referrals electronically between visits	Yes	74.7	61.9
	No	20.6	25.6
	Not Available	4.7	12.5
Safe Surgery Checklist Use	Yes	88.5	79.1
	No	6.9	8.9
	Not Available	4.6	12.0
Cardiac surgery registry	Does not have a Cardiac Surgery	40.7	55.4
	Yes	52.0	22.7
	No	3.1	1.3
	Not Available	4.2	20.7
General surgery registry	Yes	28.9	13.9
	No	66.9	65.5
	Not Available	4.2	20.7
Nursing care registry	Yes	63.9	35.4
	No	31.9	44.0
	Not Available	4.2	20.7
Stroke care registry	Yes	65.1	35.9
	No	30.7	43.4
	Not Available	4.2	20.7

^a^Based on the Hospital Compare database (N = 4861)

As displayed in Table 2, the mean length of the comments was 62.33 words (SD 63.17), wherein positive comments (37.63; SD 37.23) were significantly shorter than neutral (73.40; SD 73.03) or negative (74.34; SD 69.02) comments (*p* < 0.001). In total, 3453 topics were mentioned within the comments, whereby negative descriptions (62.4%) were more likely than positive (34.5%) or neutral (3.2%) descriptions. We classified 32.2% of all comments as overall positive, 6.0% as neutral and 61.8% as negative (inter-rater agreement: 0.793; 95 CI: 0.763–0.824).

**Table 2:** Descriptive analysis of the narrative comments and the sentiment

Overall rating result (scaled-survey ratings)^a^	Length of comments (in words)		Number of topics mentioned			Sentiment of topics (*N* = 3453; in %)				Overall sentiment of comments (*N* = 1000; in %)			
	Mean ± SD	*p* ^1^	Total	Mean ± SD	*p* ^1^	Positive	Neutral	Negative	*p* ^2^	Positive	Neutral	Negative	*p* ^2^
One star	73.02 ± 78.04	<.001	697	3.48 ± 2.15	.209	1.6	1.9	96.6	<.001	1.0	1.0	98.0	<.001
Two stars	79.05 ± 69.51		735	3.68 ± 1.17		4.1	1.4	94.6		1.5	1.0	97.5	
Three stars	72.62 ± 65.17		700	3.50 ± 2.05		14.6	6.3	79.1		2.5	12.5	85.0	
Four stars	48.53 ± 45.09		653	3.27 ± 2.11		61.1	6.1	32.8		57.5	15.0	27.5	
Five stars	38.45 ± 39.54		668	3.34 ± 2.10		97.2	0.3	2.5		98.5	0.5	1.0	
*Overall*	*62.33 ± 63.17*		*3453*	*3.45 ± 2.10*		*34.5*	*3.2*	*62.4*		*32.2*	*6.0*	*61.8*	

^a^A higher number of stars indicate a better overall rating; Note: each rating result group contained 200 analysed narrative comments

^1^Kruskal Wallis test

^2^Chi-square test

Regarding the twenty most frequently mentioned topics (see Table 3), most comments contained a description of the general impression of the patient’s hospital stay (583 out of 1000). Therein, comments were more likely to be negative (54.5%) than positive (41.0%). As demonstrated, the distribution of the topics varies among the scaled survey rating results. For example, patients were most likely to report on unintended consequences in one or two star ratings (33.7% and 30.1%, respectively), but not in very positive ratings.

**Table 3:** Results from the sentiment analysis and the distribution among the five quantitative rating categories

Nr	Category	N	Sentiment analysis of comments			Quantitative RateMDs Overall Ratings				
			Positive	Neutral	Negative	One star	Two stars	Three stars	Four stars	Five stars
1	General Impression of the care received	583	41.0%	4.5%	54.5%	21.3%	19.2%	16.0%	19.4%	24.2%
2	Demeanor Staff/Overall	248	46.8%	2.0%	51.2%	20.6%	17.7%	16.1%	18.5%	27.0%
3	Demeanor Nursing	168	36.9%	3.6%	59.5%	21.4%	21.4%	19.6%	19.6%	17.9%
4	Recommendation	152	21.7%	0.7%	77.6%	36.2%	23.7%	17.8%	5.9%	16.4%
5	Cleanliness of the facility	143	37.8%	2.8%	59.4%	32.2%	16.1%	14.0%	16.8%	21.0%
6	Demeanor Physicians	130	33.8%	1.5%	64.6%	21.5%	20.8%	22.3%	19.2%	16.2%
7	Wait time within hospital	127	7.1%	0.8%	92.1%	16.5%	33.9%	26.0%	16.5%	7.1%
8	Effectiveness of the hospital care	124	27.4%	3.2%	69.4%	22.6%	20.2%	21.0%	17.7%	18.5%
9	General Physicians	122	63.9%	7.4%	28.7%	19.7%	9.8%	17.2%	24.6%	28.7%
10	General Nursing	105	65.7%	12.4%	21.9%	7.6%	12.4%	20.0%	38.1%	21.9%
11	General Staff/Overall	102	67.6%	3.9%	28.4%	7.8%	8.8%	10.8%	28.4%	44.1%
12	Effectiveness of the staff care	95	15.8%	0.0%	84.2%	25.3%	31.6%	18.9%	12.6%	11.6%
13	Staff prompt Staff/Overall	89	40.4%	1.1%	58.4%	10.1%	25.8%	20.2%	19.1%	24.7%
14	Service	85	43.5%	2.4%	54.1%	10.6%	23.5%	18.8%	20.0%	27.1%
15	Unintended consequences	83	0.0%	0.0%	100.0%	33.7%	30.1%	26.5%	9.6%	0.0%
16	Facility Building	76	46.1%	7.9%	46.1%	14.5%	11.8%	23.7%	28.9%	21.1%
17	Care Effectiveness Physicians	73	12.3%	1.4%	86.3%	30.1%	26.0%	24.7%	13.7%	5.5%
18	Amount of Costs	57	1.8%	3.5%	94.7%	17.5%	26.3%	36.8%	17.5%	1.8%
19	Coordination of Care	54	16.7%	1.9%	81.5%	16.7%	38.9%	18.5%	11.1%	14.8%
20	Food	53	45.3%	3.8%	50.9%	13.2%	9.4%	15.1%	32.1%	30.2%

### Association between scaled survey ratings and quality of care measures

Table 4 shows the distribution of the nominally scaled clinical performance results across the scaled survey rating categories on RateMDs. Therein, the probability of choosing a high-performing hospital is greater in five star ratings compared with one star ratings in only two of the nine measures (i.e., central line-associated bloodstream infections, rate of readmission after discharge from hospital).

**Table 4:** Distribution of the nominally scaled clinical performance results according to the online ratings on RateMDs

	*Scaled survey ratings*						*Narrative comment sentiment*			
	One star	Two stars	Three stars	Four stars	Five stars	*p*	*Positive*	*Neutral*	*Negative*	*p*
Healthcare Associated Infections										
Central Line-Associated Bloodstream Infection (CLABSI)										
Better than the US national benchmark	20.9%	24.5%	22.7%	22.1%	25.7%	**	23.1%	12.3%	25.5%	**
No different than the US national benchmark	49.4%	37.2%	40.3%	39.7%	20.4%		42.3%	39.5%	26.7%	
Worse than the US national benchmark	0.6%	0.0%	1.1%	0.0%	0.0%		0.4%	1.2%	0.0%	
Number of cases too small	1.2%	0.5%	3.3%	1.0%	6.1%		2.0%	1.2%	4.1%	
Not available	27.9%	37.8%	32.6%	37.2%	47.8%		32.2%	45.7%	43.8%	
Catheter-Associated Urinary Tract Infections (CAUTI)										
Better than the US national benchmark	8.1%	8.2%	9.9%	8.1%	4.8%	**	9.1%	7.4%	5.8%	**
No different than the US national benchmark	49.4%	40.8%	45.9%	40.6%	30.6%		44.9%	35.8%	35.4%	
Worse than the US national benchmark	15.7%	16.3%	12.2%	14.7%	11.8%		15.1%	12.3%	12.2%	
Number of cases too small	1.2%	0.5%	3.3%	1.0%	6.1%		2.0%	1.2%	4.1%	
Not available	25.6%	34.2%	28.7%	35.5%	46.7%		28.8%	43.2%	42.6%	
MRSA blood Laboratory-identified Events (bloodstream infections)										
Better than the US national benchmark	4.1%	2.6%	1.7%	1.0%	1.3%	**	2.9%	1.3%	1.2%	*
No different than the US national benchmark	53.5%	50.3%	48.6%	52.5%	41.0%		50.2%	48.8%	45.9%	
Worse than the US national benchmark	3.5%	2.6%	7.2%	3.0%	0.9%		4.7%	1.3%	1.7%	
Number of cases too small	1.7%	0.0%	3.3%	1.0%	5.2%		2.2%	0.0%	3.5%	
Not available	37.2%	44.6%	39.2%	42.4%	51.5%		40.0%	48.8%	47.7%	
Clostridium difficile (C.diff.) Laboratory identified Events										
Better than the US national benchmark	19.8%	16.4%	17.7%	13.2%	14.4%	**	17.5%	13.8%	14.2%	**
No different than the US national benchmark	42.4%	48.2%	56.9%	54.3%	42.4%		52.2%	56.3%	41.0%	
Worse than the US national benchmark	15.7%	7.7%	6.6%	11.7%	7.0%		10.2%	6.3%	9.3%	
Number of cases too small	1.7%	0.0%	3.3%	1.0%	5.2%		2.2%	0.0%	3.5%	
Not available	20.3%	27.7%	15.5%	19.8%	31.0%		18.0%	23.8%	32.0%	
Readmissions Complications and Deaths										
Serious blood clots after surgery										
Better than the US national benchmark	6.4%	7.7%	6.1%	3.5%	5.2%	*	6.9%	3.8%	4.1%	
No different than the US national benchmark	62.6%	52.6%	60.6%	61.1%	47.4%		58.3%	55.0%	53.3%	
Worse than the US national benchmark	7.6%	10.7%	8.9%	8.6%	8.7%		9.5%	7.5%	8.7%	
Number of cases too small	1.2%	0.0%	3.9%	1.0%	5.7%		2.0%	1.3%	3.8%	
Not available	22.2%	29.1%	20.6%	25.8%	33.0%		23.3%	32.5%	30.1%	
Accidental cuts and tears from medical treatment										
Better than the US national benchmark	4.1%	3.1%	2.8%	4.5%	3.9%	*	3.3%	2.5%	4.7%	
No different than the US national benchmark	65.1%	60.5%	66.7%	62.6%	48.7%		63.9%	58.8%	53.8%	
Worse than the US national benchmark	7.6%	7.2%	6.7%	6.1%	7.8%		7.3%	6.3%	7.0%	
Number of cases too small	1.2%	0.0%	3.9%	1.0%	5.2%		2.2%	0.0%	3.5%	
Not available	22.1%	29.2%	20.0%	25.8%	34.3%		23.3%	32.5%	31.1%	
Collapsed lung due to medical treatment										
Better than the US national benchmark	0.6%	1.0%	0.6%	0.5%	0.4%	*	0.9%	1.3%	0.3%	
No different than the US national benchmark	74.3%	66.3%	75.0%	71.6%	59.1%		71.8%	65.0%	64.2%	
Worse than the US national benchmark	1.8%	3.6%	1.1%	1.0%	2.2%		2.0%	1.3%	1.7%	
Number of cases too small	1.2%	0.0%	3.9%	1.0%	5.2%		2.2%	0.0%	3.5%	
Not available	22.2%	29.1%	19.4%	25.9%	33.0%		23.1%	32.5%	30.2%	
Serious complications (PSI-90-SAFETY)										
Better than the US national benchmark	3.5%	2.6%	3.3%	5.1%	3.0%	*	3.3%	1.3%	4.1%	
No different than the US national benchmark	66.3%	60.2%	68.0%	61.6%	50.4%		64.5%	58.8%	54.9%	
Worse than the US national benchmark	7.0%	8.2%	5.5%	6.6%	7.0%		7.1%	7.5%	6.4%	
Number of cases too small	1.2%	0.0%	3.3%	1.0%	5.2%		1.8%	0.0%	3.5%	
Not available	22.1%	29.1%	19.9%	25.8%	34.3%		23.3%	32.5%	31.1%	
Rate of readmission after discharge from hospital (hospital-wide)										
Better than the US national benchmark	10.4%	9.2%	14.9%	10.6%	15.7%	*	11.7%	8.8%	14.0%	
No different than the US national benchmark	72.3%	70.4%	68.5%	71.7%	70.7%		70.9%	73.8%	69.8%	
Worse than the US national benchmark	15.0%	14.3%	9.9%	16.2%	8.3%		12.8%	11.3%	12.8%	
Number of cases too small	0.0%	5.6%	3.3%	0.0%	2.2%		2.4%	5.0%	1.5%	
Not available	2.3%	0.5%	3.3%	1.5%	3.1%		2.4%	1.3%	2.0%	

Chi-square test (* *p* < 0.05; ** *p* < 0.001)

After adjusting for hospital characteristics, we detected significant associations between the metrically scaled quality measures and the scaled online ratings for thirteen of the twenty-nine quality of care measures (Table 5). Regarding healthcare associated infections, central line-associated bloodstream infections were negatively associated with the rating results on RateMDs; the higher the number of stars of the rating (i.e., the better the rating), the lower the infection scores (*ρ* = − 0.087, *p* < 0.05). Further significant associations were measured between the scaled online ratings and two readmission, complications and deaths measures. Interestingly, the results for the measure collapsed lung due to medical treatment were positively associated with the ratings (*ρ* = 0.080, *p* < 0.05), whereas lower readmission rates after discharge from hospital were negatively associated with the online ratings (*ρ* = − 0.070, *p* < 0.05). Finally, the associations between online ratings and timely and effective care measures proved to be significant in ten of the twenty measures (*ρ* = ±0.143, *p* < 0.05 for all), wherein six significant associations indicate better clinical outcomes for higher star ratings. In sum, eight out of the thirteen determined significant associations indicate better clinical outcomes for higher star ratings.

**Table 5:** The association between online ratings and quality of care measures (Spearman rank coefficient of correlation)

Clinical quality of care measures		N	Adjusted association with scaled survey ratings (*p* value)^a^		Adjusted association with narrative comment sentiment (*p* value)^a^	
Healthcare Associated Infections						
1	HAI_1_SIR: Central Line-Associated Bloodstream Infection (CLABSI)	829	−0.087	.012	−0.061	.080
2	HAI_2_SIR: Catheter-Associated Urinary Tract Infections (CAUTI)	862	0.018	.587	0.035	.302
3	HAI_5_SIR: MRSA blood Laboratory-identified Events (bloodstream infections)	748	−0.017	.648	−0.027	.463
4	HAI_6_SIR: C. diff. Laboratory identified Events (Intestinal infections)	959	−0.061	.053	0.016	.608
Readmissions Complications and Deaths						
5	PSI_12_Score: Serious blood clots after surgery	941	0.035	.282	0.039	.230
6	PSI_15_Score: Accidental cuts and tears from medical treatment	937	−0.008	.799	−0.005	.871
7	PSI_6_Score: Collapsed lung due to medical treatment	944	0.080	.013	0.078	.016
8	PSI_90_Score: Serious complications (summary measure; PSI-90-SAFETY)	938	0.019	.559	0.021	.509
9	READM_30_Score: Rate of readmission after discharge from hospital	982	−0.070	.015	0.011	.700
Timely and Effective Care						
10	ED_1b: Average time patients spent in the emergency department, before they were admitted to the hospital as an inpatient	950	−0.052	.100	−0.052	.103
11	ED_2b: Average time patients spent in the emergency department, after the doctor decided to admit them as an inpatient before leaving the emergency department for their inpatient room	949	−0.038	.228	−0.051	.112
12	OP_18b: Average time patients spent in the emergency department before being sent home	930	−0.094	.004	−0.085	.008
13	OP_20: Average time patients spent in the emergency department before they were seen by a healthcare professional	928	−0.090	.005	−0.113	.000
14	OP_22: Percentage of patients who left the emergency department before being seen	942	−0.096	.002	−0.058	.059
15	OP_6: Outpatients having surgery who got an antibiotic at the right time - within one hour before surgery	927	−0.083	.009	−0.034	.284
16	OP_7: Outpatients having surgery who got the right kind of antibiotic	927	0.061	.056	0.041	.201
17	SCIP_CARD_2: Surgery patients who were taking heart drugs called beta blockers before coming to the hospital, who were kept on the beta blockers during the period just before and after their surgery	971	−0.010	.738	−0.059	.053
18	SCIP_INF_1: Surgery patients who were given an antibiotic at the right time (within one hour before surgery) to help prevent infection	979	−0.066	.026	−0.026	.380
19	SCIP_INF_10: Patients having surgery who were actively warmed in the operating room or whose body temperature was near normal by the end of surgery	984	−0.065	.027	−0.079	.007
20	SCIP_INF_2: Surgery patients who were given the right kind of antibiotic to help prevent infection	979	0.049	.099	−0.023	.429
21	SCIP_INF_3: Surgery patients whose preventive antibiotics were stopped at the right time (within 24 h after surgery)	979	−0.013	.672	−0.098	.001
22	SCIP_INF_9: Surgery patients whose urinary catheters were removed on the first or second day after surgery.	977	0.037	.213	−0.011	.709
23	SCIP_VTE_2: Patients who got treatment at the right time (within 24 h before or after their surgery) to help prevent blood clots after certain types of surgery	980	−0.097	.001	−0.114	.000
24	VTE_1: Patients who got treatment to prevent blood clots on the day of or day after hospital admission or surgery	967	0.143	.000	0.070	.028
25	VTE_2: Patients who got treatment to prevent blood clots on the day of or day after being admitted to the intensive care unit (ICU)	940	0.058	.073	0.034	.296
26	VTE_3: Patients with blood clots who got the recommended treatment, which includes using two different blood thinner medicines at the same time	918	0.111	.001	0.049	.133
27	VTE_4: Patients with blood clots who were treated with an intravenous blood thinner, and then were checked to determine if the blood thinner was putting the patient at an increased risk of bleeding	779	0.009	.797	0.001	.991
28	VTE_5: Patients with blood clots who were discharged on a blood thinner medicine and received written instructions about that medicine	907	0.038	.256	0.006	.864
29	VTE_6: Patients who developed a blood clot while in the hospital who did not get treatment that could have prevented it	602	−0.117	.007	−0.054	.187

^a^Adjusted for Hospital Type, Hospital Ownership, and Emergency Service

Abbreviations: PSI: Patient Safety Indicators, ED: Emergency Department, OP: Outpatient, SCIP: Surgical Care Improvement Project, VTE: Venous Thromboembolism

### Association between narrative comments and quality of care measures

When choosing a hospital based on the sentiment of the narrative comments, the probability of selecting a high-performing hospital is greater in five of the nine measures (see Table 4). However, we could also detect (in most cases marginally) higher percentages for selecting a low-performing hospital for narrative comments with a positive sentiment in eight of the nine measures. After adjusting for hospital characteristics, seven of the twenty-nine metrically scaled clinical measures were significantly associated with the sentiment of the patient narratives (Table 5). In line with the results above, narrative comments are negatively associated with one readmission, complication and death measure (collapsed lung due to medical treatment; *ρ* = 0.078, *p* < 0.05), indicating lower clinical performance scores in narrative comments with a positive sentiment. The significant associations between the comments and timely and effective care measures were determined to be inconsistent. Three associations indicate better clinical outcomes in more favorable narrative comments: (1) average time patients spent in the emergency department before being sent home (*ρ* = − 0.085, *p* < 0.05); (2) average time patients spent in the emergency department before they were seen by a health care professional (*ρ* = − 0.113, *p* < 0.001); and (3) patients who got treatment to prevent blood clots on the day of or day after hospital admission or surgery (*ρ* = 0.070, *p* < 0.05). In contrast, three quality measures are negatively associated with the sentiment of the comments: (1) patients having surgery who were actively warmed in the operating room or whose body temperature was near normal by the end of surgery (*ρ* = − 0.079, *p* < 0.05); (2) surgery patients whose preventive antibiotics were stopped at the right time (within 24 h after surgery) (*ρ* = − 0.098, *p* < 0.05); and (3) patients who got treatment at the right time (within 24 h before or after their surgery) to help prevent blood clots after certain types of surgery (*ρ* = − 0.114, *p* < 0.05). Finally, we determined a significant correlation between the scaled survey online ratings and the sentiment of the narrative comments (*ρ* = 0.797; *p* < 0.001).

## Discussion

This study determined the association between online ratings and clinical quality of care measures to assess the usefulness of the ratings for patients when searching for a hospital. In contrast to previous studies (see below), we collected an equal number of very positive, positive, neutral, negative and very negative online ratings to get a more in-depth knowledge of the association and distribution of the online ratings according to the clinical performance. Our results show that online ratings seem to have limited potential to guide patients to high-performing hospitals. Based on our analysis, relying on a very positive online rating was associated with a higher probability of selecting a high-performing hospital in only two of the nine nominally scaled measures. Furthermore, the probability of selecting such a hospital was greatest in very negative online ratings in two measures. We further present some modest associations between metrically scaled online ratings and clinical performance measures (*ρ* = ±0.143, *p* < 0.05 for all). Therein, eight of the thirteen significant associations indicate better clinical outcomes for higher star ratings.

We could detect a significant association between the general rate of readmission after discharge from hospital (*ρ* = − 0.070, *p* < 0.05) and the online ratings, which is in line with the results from two similar studies. First, the authors showed slightly stronger, but still weak and modest significant correlations between scaled survey online ratings for US hospitals from Yelp and three readmission related outcome measures (myocardial infarction, − 0.17; heart failure, − 0.31; pneumonia, − 0.18) and two of three mortality outcome measures (myocardial infarction, − 0.19; pneumonia, − 0.14) [23]. Second, the study from the UK showed mixed but also slightly stronger results [37]. While positive online recommendations displayed on NHS Choices were significantly associated with lower hospital standardized mortality ratios (*ρ* = − 0.20; *p* = 0.01), lower mortality from high-risk conditions (*ρ* = − 0.23; *p* = 0.01), and lower readmission rates within 28 days (*ρ* = − 0.31; *p* < 0.001), no association could be determined with mortality rates among surgical inpatients with serious treatable complications (*ρ* = 0.00; *p* = 0.99) or mortality from low-risk conditions (*ρ* = 0.03; *p* = 0.70). The results from those two studies indicate that better online ratings are associated with better clinical outcomes. Whether the fact that the authors used disease-specific performance metrics for their analysis might account for the stronger associations should be addressed in future research.

However, our results also indicate that better online ratings can be associated with lower clinical outcomes. One reason for this finding might be that the rating system of RateMDs does not explicitly cover certain aspects of clinical care or the quality of the care process. Looking at those clinical indicators for which a negative association with the online ratings could be determined, it becomes apparent that those are hardly covered by any of the RateMDs rating categories. For example, three negative associations were related to receiving care at the right time (e.g., outpatients having surgery who got an antibiotic at the right time—within one hour before surgery). It seems likely that patients might not be able to capture or be even aware of the time an antibiotic or similar treatment has to be given.

The evidence regarding the association between narrative comments and clinical performance measures is even more scarce. As shown above, relying on comments with a positive sentiment leads to a higher probability of choosing a well-performing hospital in five of the nine nominally scaled measures. However, this choice behavior would also increase the risk of choosing a low-performing hospital. Furthermore, only seven of the twenty-nine associations between the sentiment of the narrative comments and metrically scaled clinical outcomes could be shown to be statistically significant. Therein, three associations indicate better clinical outcomes in more favorable narrative comments whereas four measures indicate lower clinical performance scores in positive narrative comments. Consequently, it might be questionable whether their broader incorporation into report cards would be of use for patients [22]. Also interestingly, quality measures for which a significant association with the sentiment of the comments could be detected were all but one (i.e., surgery patients whose preventive antibiotics were stopped at the right time-within 24 h after surgery)—the same indicators for which the quantitative ratings have also shown a significant association. In addition, all of them showed the same direction of association. Given the fact that users are also only able to process a limited amount of information at a time so as not to be overwhelmed with information [38], it seems reasonable to state that the information that contributes to better decisions (e.g., in terms of selecting a high-performing hospital) should be particularly presented [39]. Taken together, despite recent suggestions of incorporating narrative comments into report cards [14, 33, 40–42], their usefulness in the report card’s current form might be limited for patients who search for a well performing hospital.

One possible reason for this might be the request posed for leaving a comment on the report card RateMDs which served as the basis for our analysis, which is as follows: “Please leave a comment with more detail about your experience.” The fact that it does not seem to be very specific might account for the fact that more general comments were being left by the patients. It may be possible that narrative comments from other report cards lead to different findings, even though the posed requests there do not seem to be not much more specific, as the following examples demonstrate: “Your review: Your review helps others learn about great local businesses” (Yelp); “Write your review: Add a review” (beside it for a few seconds appears: “A good review is: both detailed and specific; Consider writing about: pros and cons, some things people might not know about the listing”) (Find the Best – Health Grove); and “Write a Review” (Wellness). Whether or not narrative comments from those rating websites lead to different findings shall be addressed in future studies. In addition, research should also assess whether more specific requests would lead to comments which are more highly correlated with clinical performance metrics and thus might add value for patients when searching for a well performing hospital.

As mentioned above, a recent study from the Netherlands has shown that low online ratings might be used so that patients avoid low-performing hospitals [20]. More specifically, the authors have demonstrated that information from social media can be used to integrate patient’s perspective in supervision and this information could be used from health care inspectorates to undertake risk-based supervision of elderly care. Based on this, we analyzed whether low online ratings from RateMDs might be helfpul for patients so as to avoid low-performing hospitals. When looking at the distribution of the one star scaled survey ratings on RateMDs, we could see similar percentages for low and high performing hospitals (Better than the US national benchmark: 8.7% vs. Worse than the US national benchmark: 8.3%, respectively). Here, most hospitals can be assigned to the average performance group (No different than the US national benchmark: 59.5%). Based on those numbers, we conclude that low online ratings are of limited usefulness for patients when trying to avoid low-performing hospitals. However, further research should explore the usefulness of low online ratings more in detail.

There are some limitations that have to be taken into account when the results of this study are interpreted. First, our study adopted a cross-sectional design, so we were able to identify associations between exposure and outcomes but could not infer cause and effect. Second, our systematic search procedure was limited to the Medline database (via PubMed). We did not include further databases since it was not our primary aim to carry out a comprehensive and systematic literature review but to capture the literature in the most relevant database. However, we checked all references in the studies and also searched Google to capture relevant literature. Due to our different approach by incorporating an equal number of ratings among all rating scores, our results should be compared with caution with those from other studies. For example, it is not surprising that the percentage of narrative comments with a negative sentiment is larger in our study compared with previously published studies, since most ratings on report cards have been shown to be mostly positive (see above). Furthermore, since one purpose of this study was to address the differences of ratings among the five rating scores we did not create a representative sample of hospitals. Nevertheless, as shown above we calculated risk-adjusted result. In addition, it should be mentioned that we did not analyze the validity or reliability of the used quality indicators. Instead, we used those quality indicators that are being published on the report card Hospital Compare. As a further limitation, it has to be mentioned that our study determined the usefulness of online ratings for patients when searching for a hospital by assessing the association between online ratings and clinical quality of care measures. Nevertheless, research has demonstrated that patients might prefer other aspects of care when choosing a hospital [43]. The analysis of the association of such measures and online ratings might lead to different findings. Finally, our analysis is only based on online ratings from the US report card RateMDs. Thus, our findings cannot be generalized for online ratings on other US hospital rating websites or those from other countries. The analysis of ratings from other US websites might lead to other conclusions. In addition, it might be interesting to compare the narratives between report cards from different countries. Because of the major differences between the systems in the US and other countries, there might be also differences in the way patient rate and tell their story about hospitals.

## Conclusions

In sum, whether patients who search for a well performing hospital in terms of clinical quality of care should rely on online ratings to make a choice can be answered in part. Based on our results, there seems to be some association between quantitative online ratings and clinical performance measures. Nevertheless, the relatively weak strength and inconsistency of the direction of the associations, as well as the lack of association with several other clinical measures, may not enable us to draw strong conclusions. For some measures, we even detected a negative association, which has the potential to mislead patients. Despite the promise of incorporating narrative comments into report cards to engage patients in their use, they seem to have limited potential to reflect the clinical quality of care in its current form. Only a small proportion of the tested associations was statistically significant; four out of the seven were even negatively associated with the sentiment of the comments. In addition, the indicators for which a significant association with the sentiment of the comments could be detected were almost all covered by indicators for which the quantitative ratings had shown a significant association. Whether or not the usefulness of narrative comments can be increased by posing more specific requests for leaving a narrative comment should be addressed in future studies.

## Additional file

Additional file 1: Word document. Overview of the applied categorization scheme & Codebook. (DOCX 42 kb)

## Abbreviations

C. diff.: Clostridium difficile
CAUTI: Catheter-Associated Urinary Tract Infections
CLABSI: Central Line-Associated Bloodstream Infection
CMS: Centers for Medicare and Medicaid Services
MRSA: Methicillin-resistant *Staphylococcus aureus*
NHS: National Health Service
UK: United Kingdom
US: United States of America
