# Supplementary material for: A cross-sectional study assessing the association between online ratings and clinical quality of care measures for US hospitals: results from an observational study
Source: BMC Health Serv Res. 2018 Feb 5;18:82. doi: 10.1186/s12913-018-2886-3 (PMC5800028; doi:10.1186/s12913-018-2886-3)
Supplement: Additional file 1: — Word document. Overview of the applied categorization scheme & Codebook. (DOCX 42 kb) [file 12913_2018_2886_MOESM1_ESM.docx]

**Overview of the applied categorization scheme**

1. Care
   1. General impression of the received care
   2. Service
   3. Access
   4. Effectiveness
      1. Effectiveness of the hospital care
      2. Effectiveness of the staff care
      3. Care Effectiveness Physicians
      4. Care Effectiveness Nursing
      5. Care Effectiveness Others
   5. Unintended consequences
   6. Coordination of Care
2. Facility
   1. Cleanliness of the facility
   2. Facility Building
   3. Rooms (patient room, examination room)
   4. Medical equipment
   5. Parking/transportation
   6. Noise
   7. Food
3. Wait time
   1. Wait time within hospital
   2. Wait time outside hospital
4. Clinicians and Staff
   1. Service (Staff/Overall, physicians, nursing, administration, others)
   2. Hygiene Service (Staff/Overall, physicians, nursing, administration, others)
   3. Staffing Level (Staff/Overall, physicians, nursing, administration, others)
   4. Staff Prompt (Staff/Overall, physicians, nursing, administration, others)
   5. Demeanor (Staff/Overall, physicians, nursing, administration, others)
   6. Ability (Staff/Overall, physicians, nursing, administration, others)
   7. General (Staff/Overall, physicians, nursing, administration, others)
   8. Availability (Staff/Overall, physicians, nursing, administration, others)
   9. Communication (Staff/Overall, physicians, nursing, administration, others)
5. Communication (Others)
   1. Scheduling
   2. Complaint Management
6. Costs
   1. Amount of Costs
   2. Only focused on money
   3. Insurance related issue
   4. Proper/Wrong billing
   5. Early/Late billing
7. Personal issues
   1. Privacy
   2. Patient rights
8. Acknowledgements (Hospital, staff/overall, physicians, nursing, others)
9. Recommendation
10. Other comments

**Codebook**

| Category | Example: Positive concern | Example: Negative concern | Category Description |
| --- | --- | --- | --- |
| 1. Care | The following categories contain information about the provided care and general impressions of the hospital. | | |
| 1.1 General impression of the received care | “…best hospital in the world…”; “…it was a great experience...” | “…worse hospital I've every been to in my whole life…”; “…This hospital was one of the worst you have ever seen, and after this length of time it's even gotten worse…” | This category describes the general impression towards the hospital and the received care. |
| 1.2 Service | “very good in all the services”; “…Great service…” | “…Very poor service along the way…”; “…lowest class service…” | The category “service” characterizes the service of the hospital. |
| 1.3 Access | “I was admitted quickly…”; “…Admission was professional and fast…” | “…Only one problem I found, which was admission. It takes a long time to get through all the paperwork…”; “…Central admission is required for even the simplest and safest procedure. Forms and questions seem not to vary by procedure. Some, but not all, relevant data is recalled from previous admissions. A subsidiary, Sugar Land Surgical Center, handles admission much better by allowing on-line registration…” | This category describes the access/admission procedure in the hospital. For example, it explains the speed of admission or the willingness to provide treatments. |
| 1.4. Effectiveness | The following categories characterize the quality of care related to different hospital employees. | | |
| 1.4.1 Effectiveness of the hospital care | “…Pain management was a priority as well…”; “…I was given all appropriate tests, vital signs monitored…” | “…my mother was pushed out too soon.”; “…No testing, no diagnostics, sent me home with a kidney stone…” | This category describes the quality of treatments or the quality of care related to the hospital. |
| 1.4.2 Effectiveness of the staff care | “…They all took great care of him. It was diagnosed as bronchitis as EKG was clear…”; “…They see you in a reasonable amount of time (Quickly!), and that's really important when you're hurting. My problem was ALWAYS treated properly…” | “…(1)our 15 year old son had surgery; when quitting time came, ALL of the staff on the outpatient floor left, leaving our son with his IV still in his arm, hallucenating (they overdosed him on phenegrin)…”; “…They will throw you out before your are stable enough to go home. I couldn't believe it when i had abdominal surgery one evening and they sent me home the next morning without me having had gas or a bowel movement…” | This category describes the quality of treatments or the quality of care related to the staff. |
| 1.4.3 Effectiveness of the physicians’ care | “…Dr Humphries and his team fixed my neck (A+)…”; “…This doctor performed an orbital decompression on me and totally corrected the problem…” | “My left foot was swollen and very painful. Went to ER @St. Lukes and was x-rayed. Dr said no broken bones -- probably cellulitus. However, I went to my podiatrist after 2 weeks, because of continued pain and he took an x-ray and it was broken…”; “…The surgeon was cold, rude, literally walked out of the room when I was in the middle of a question. He attempted to discharge me mere hours after an invasive procedure that typical puts people in the hospital for 24-48 hours. He cut me off IV pain meds, put me on a oral pain killer than did nothing for me…” | This category describes the quality of treatments or the quality of care related to the physicians. |
| 1.4.4 Effectiveness of the care of nurses | “…The doctors, nurses, and all the staff cared about me, my family, and providing me with an excellent outcome…”; “…All the nurses that I came in contact with were wonderful as were the doctors. I have not always been able to say that about other hospitals. They kept the pain under control and I can't think of one complaint…” | “…The nurse gave me the wrong dose of a medication and I had a seizure…”; “…Nurses lost one of my meds. Stood there and overheard nurse telling another patient they couldn't find they're meds! Had to tell them how I was taking my meds SEVERAL times. What I actually DID and Did not take. Have to basically BEG to get something to take for a Headache and waite 1/2 to a day and 1/2 to get 2 tylenol…” | This category describes the quality of treatments or the quality of care related to the nurses. |
| 1.4.5 Effectiveness of the care of others | “…she has help me with a cough that i had for over 9 years and no one could help me. Allergy are much better and my cough is now control and so bad like it was…” | “…DO NOT LET AN ANESTHESIOLOGY STUDENT PUT THE EPIDURAL. This "student" screwed me over". I have nerve damage from that, and now I have tingling,pins and needles sensation on my arms, hands,legs…”; “This hospital allowed a resident to operate on me without my consent!!! He COMPLETELY LACERATED MY EHL TENDON IN MY BIG TOE…” | This category describes the quality of treatments or the quality of care related to other hospital employees. |
| 1.5 Unintended consequences |  | “…Myself and a family memeber almost died from infections I was told that was caught in the hospital…”; “…Each and every time I get a random nurse who seemingly doe not know how to draw said blood, and have had a few prick my arms no less than 4 times because they just couldn't hit my vein. I've gotten bruises along my arms from this….” | The category “Unintended consequences” characterizes the occurred consequences due to malpractice in the hospital. |
| 1.6 Coordination of Care | “… coordinated.”; “…Appropriate steps seem to be taken in an organized, efficient manner.” | “…We feel staff is either inept or they are so uncaring and disorganized…”; “This hospital is extremely disorganized…” | This category explains how the hospital care is coordinated and how the service providers are communicating with each other. |
| 2. Facility | The following categories are addressing topics related to the facility. | | |
| 2.1 Cleanliness of the facility | “…The hospital is very clean…”; “…facility was clean…” | “Not very clean…”; “The floors are filthy…” | The category “cleanliness” describes if the hospital is in a clean condition. |
| 2.2 Facility Building | “…modern facilty…”; “…facility lovely…” | “…I recommend this hospital. It is not the newest…”; “…old facility…” | This category characterizes the condition of the facility due to appearance and construction. |
| 2.3 Rooms | This category gives information about the hospital rooms. It distinguishes between patient and examination rooms. | | |
| 2.3.1 Patient rooms | “Private rooms are nice…”; “…my room was brand new…” | “…the room was dirty and the chair was ripped…”; “…none of the rooms are modern or comfortable…” | This category explains the appearance of the patient rooms and the related equipment. |
| 2.3.2 Examination rooms | “Beautiful brand new ER and OR's…”; “…I proceeded immediately to one of the ER rooms(seperate room for each patient which is great)…” | “…The test prior to back surgery to determine extent of loss of feeling in legs was performed here but in a junk closet with hooks boxes and clutter everywhere…”; “…but Recovery room was VERY Poor…” | This category explains the appearance of the examination rooms and the related equipment. |
| 2.4 Medical equipment | “…latest equipment…”; “While Lourdes is a gorgeous facility, with outstanding equipment…” | “…Testing equipment breaks down on a regular basis…”; “…they must have only one stress EKG in entire hospital…” | The category “medical equipment” describes the condition, the state-of-the-art and the availability of the equipment in the hospital. |
| 2.5 Parking/transportation | “…Great parking (even for bigger vehicles)...”; “…easy parking…” | “…Parking inadequate.”; “The parking may be a bit difficult during busy days…” | This category characterizes the parking or transportation situation of the hospital. |
| 2.6 Noise | “…The unit was immaculate, quiet, and private, and modern…”; “…hospital is quiet…” | “…hallway noisy all night…”; “It is little noisy…” | The category “noise” explains the noise level in the hospital. |
| 2.7 Food | “…The food was great came on time…”; “…Meals were wellprepared and served hot…” | “…food - not fit for human consumption…”; “…the food was uneatable…” | This category comprises the taste, quality and the condition of the served food. |
| 3. Wait time | The following categories give information about the wait time inside the hospital for treatments and about the wait time outside the hospital for appointments. | | |
| 3.1 Wait time within hospital | “…We weren't waiting long…”; “…minimal wait time…” | “Long wait for surgery…”; “…waiting 3-5 hours after being admitted to see a doc just seems outrageous…” | This category contains if the patient has to wait for treatment within the hospital. |
| 3.2 Wait time outside hospital |  | “I had to wait two months for an appointment with neurology for a rare nerve condition I have...”; “…Now I have to wait 2 months to be seen…” | This category contains if the patient has to wait for treatments or appointments outside the hospital. |
| 4. Clinicians and Staff | The following categories are addressing topics that belong to clinicians and staff. | | |
| 4.1 Service | This category gives general information about the provided service due to different hospital employees. | | |
| 4.1.1 Service Staff/Overall | “Excellent level of care and service by nurses and all staff.”; “Great care and services from the staff…” | “…They allowed Dr. Patrick Hitchon to paralyze me for life, then refused to release any of my medical records…”; “…No one offered a wheelchair or any assistance…PITIFUL SERVICES…” | The category explains the level of service due to the hospital staff. |
| 4.1.2 Service Physicians | “…full service by techs, nurses and doctor…” | “…the service of nurses and doctors is terrible…”; “…the service and overall treatment I received from the doctor was pretty bad…” | The category explains the level of service due to the physicians. |
| 4.1.3 Service Nursing | “…full service by techs, nurses and doctor…”; “Excellent level of care and service by nurses and all staff.” | “…the service of nurses and doctors is terrible…”; “…From the lies during the initial walk-through by Nikki Mederos to the horrible service from nurses such as Sharon Fischer, this was my worst nightmare…” | The category explains the level of service due to the nurses. |
| 4.1.4 Service Administration |  | “…A good administration can improve scheduling & efforts should be made by better, careful planning to avoid OVER utilization of the Hospital's facilities…” | The category explains the level of service due to the hospital administration. |
| 4.1.5 Service Others | “…full service by techs, nurses and doctor…” | “…Now they are refusing to send my records to my insurance company, after multiple requests from both me and my insurance…” | The category explains the level of service due to other hospital employees. |
| 4.2 Hygiene | The following categories address the sanitary behavior of different hospital employees and the hygiene standards. | | |
| 4.2.1 Hygiene Staff/Overall | “…Observed excellent hygiene standards at all levels…”; “…They are well trained, take extensive precautions to reduce staff infections…” | “…Something falls on the floor, they pick it up and use it in the patients mouth…”; “…Asking them to WASH HANDS before touching me was greeted with a blank stare. Their reply was they had used antibacterial hand cream and gloves (pulled from an open container ANYONE might have sneezed on.)…” | This category characterizes the hygiene standards and the sanitary behavior of the staff. |
| 4.2.2 Hygiene Physicians |  | “…Hand washing by “professionals” seems optional before touching patients…”; “…doctors=rarely available, do not wear gloves and do not wash hands…” | This category characterizes the hygiene standards and the sanitary behavior of the physicians. |
| 4.2.3 Hygiene Nursing |  | “…THE NURSE WHO GAVE ME TO TRANSFUSIONS PUT “STERILE” GLOVES ON THEN PULLED A DIRTY TRASH CAN OVER (WITH THE GLOVES ON) TO EMPTY OUT SOME OF THE BLOOD INTO THE TRASH CAN…”; “…She was sniffling and she sneezed into her open bare left hand. She then put her hand on the work surface of her station. She did not find purell to sanitize her hand, she continued working…” | This category characterizes the hygiene standards and the sanitary behavior of the nurses. |
| 4.2.4 Hygiene Administration |  |  | This category characterizes the hygiene standards and the sanitary behavior of the administration staff. |
| 4.2.5 Hygiene Others |  |  | This category characterizes the hygiene standards and the sanitary behavior of other hospital employees. |
| 4.3 Staffing Level | The following categories contain topics about the staffing level in the hospital due to different hospital employees. | | |
| 4.3.1 Staffing Level Staff/Overall | “…The unit was very well staffed...” | “…not enough staff to help you or monitor you…”; “…This hospital, like many others, is short staffed…” | This category informs about the staffing level of the hospital staff and describes if the hospital has recruited enough or too many employees. |
| 4.3.2 Staffing Level Physicians | “…excellent physician staffing…” | “…I think they should of spend the remodeling money on more doctor…”; “…because the hospital has very few staff doctors.” | This category informs about the staffing level of the physicians and describes if the hospital has recruited enough or too many physicians. |
| 4.3.3 Staffing Level Nursing |  | “…but the nurses and CNAs are very short-staffed…”; “…THE STAFFING LEVELS OF NURSE TO PATIENT IS SAD…” | This category informs about the staffing level of the nurses and describes if the hospital has recruited enough or too many nurses. |
| 4.3.4 Staffing Level Administration |  |  | This category informs about the staffing level of the hospital administration and describes if the hospital has recruited enough or too many employees for the administration. |
| 4.3.5 Staffing Level Others |  | “…Ask how many VP's they have for less then a 275 bed hospital....23 last count…”; “…understaffed in aides…” | This category informs about the staffing level of other hospital employees (e.g. kitchen staff) and describes if the hospital has recruited enough or too many other employees. |
| 4.4 Staff prompt | The following categories address topics that contain information about the promptness, efficiency and timeliness due to different hospital employees. | | |
| 4.4.1 Staff prompt Staff/Overall | “…They had me in a room in no time…”; “…They followed his orders immediately, even though they had to make extra phone calls.” | “…You can be on a call light for care for hours…”; “…They only start to respond when there is a clear state of emergency, and even that needs to be brought to their attention. …” | This category comprises if the staff reacts quickly to answer requests or to provide treatments. It also gives information about the efficiency and timeliness of the staff. |
| 4.4.2 Staff prompt Physicians | “…The personell; nurses, aids, doctors, etc. were efficient…”; “…The physicians came quickly…” | “…Doctor was very busy…”; “….Doctors are very hard to get to, not keeping appointments, always too busy…” | This category comprises if the physicians react quickly to answer requests or to provide treatments. It also gives information about the efficiency and timeliness of the physicians. |
| 4.4.3 Staff prompt Nursing | “…Nursing staff very knowledgeable and prompt.”; “…The personell; nurses, aids, doctors, etc. were efficient…” | “Nurses too busy…”; “…none of the nurses speak english and took her 6 hrs to get some crappy food out of them…” | This category comprises if the nurses react quickly to answer requests or to provide treatments. It also gives information about the efficiency and timeliness of the nurses. |
| 4.4.4 Staff prompt Administration |  |  | This category comprises if the administration reacts quickly to answer requests or to provide treatments. It also gives information about the efficiency and timeliness of the administration. |
| 4.4.5 Staff prompt Others | “…room service was prompt…”; “…The personell; nurses, aids, doctors, etc. were efficient…” |  | This category comprises if other hospital employees react quickly to answer requests or to provide treatments. It also gives information about the efficiency and timeliness of other employees. |
| 4.5 Demeanor | The following categories describe the behavior of different hospital employees. | | |
| 4.5.1 Demeanor Staff/Overall | “…Friendly staff…”; “…The staff was professional, courteous and extremely helpful…” | “…Staffing not very professional…”; “They dont care for the elderly they are rude” | The category “demeanor” describes the behavior of the staff towards the patients and their relatives. |
| 4.5.2 Demeanor Physicians | “…doctors were all very helpful and courteous…”; “…doctors, etc. were efficient, friendly & helpful…” | “…The surgeon was cold, rude, literally walked out of the room when I was in the middle of a question…”; “…Doctor did nothing to help me. He played with nurses instead.” | The category “demeanor” describes the behavior of the physicians towards the patients and their relatives. |
| 4.5.3 Demeanor Nursing | “…Nurses on floor were very attentive…”; “…The nursing staff was great on the floor I was on, very kind and helpful…” | “…rude nurses…”; “…the nurses were not friendly…” | The category “demeanor” describes the behavior of the nurses towards the patients and their relatives. |
| 4.5.4 Demeanor Administration |  | “…Nor will he forget the unkind hospital administrator who told us not to come back…”; “…corrupt administration” | The category “demeanor” describes the behavior of the administration towards the patients and their relatives. |
| 4.5.5 Demeanor Others | “…Security is wonderful here! Very helpful and honest when I had to retrieve my wallet and glasses!...”; “…Security guards are pleasant and helpful…” | “…As I was waking up from surgery, some large burly lady was standing over me demanding immediate payment…”; “Their billing department is horrible and the billing department staff is just plain mean and uncaring…” | The category “demeanor” describes the behavior of other hospital employees towards the patients and their relatives. |
| 4.6 Ability | The following categories are describing the education levels and skills of the different hospital employees. | | |
| 4.6.1 Ability Staff/Overall | “…The staff is educated…”; “…All the staff really know their stuff…” | “…We feel staff is either inept or they are so uncaring…”; “…The entire staff is rude and not very knowledgeable…” | This category characterizes the training/education or the knowledge of the hospital staff. |
| 4.6.2 Ability Physicians | “An unusual gap between many highly educated professionals and a business management system that burns everybody.”; “Had c-section done and the Ob-Gyn although knowelegeable was very rude to the point of being mean…” | “…Unsafe ER Doctors who can not read an X-ray & lie to you!”; “Ill trained er docs” | This category characterizes the training/education or the knowledge of the physicians. |
| 4.6.3 Ability Nursing | “…Nursing staff very knowledgeable and prompt.”; “…found the nurses to be very compassionate and off hi quality” | “…Too many student nurses that do not know what they are doing.”; “…I will spare you the rest of my medical nightmare with rude doctors and undereducated nurses…” | This category characterizes the training/education or the knowledge of the nurses. |
| 4.6.4 Ability Administration |  |  | This category characterizes the training/education or the knowledge of the administration staff. |
| 4.6.5 Ability Others | “…Blood was drawn and an Ultrasound was conducted by a kind, professional and knowledgeable technician…”; “…The receptionist fellow, was very nice and knew what he was doing…” | “…Their people took my information down wrong and charged me money to correct it.”; “…Either aides were not trained in procedure or not supervised correctly....” | This category characterizes the training/education or the knowledge of other hospital employees. |
| 4.7 General | The following categories contain general information about the different hospital employees. | | |
| 4.7.1 General Staff/Overall | “Great nurses and staff…”; “Their great staff treated me wonderfully.” | “The staff was so horrible…”; “…This is the worst and most unprofessional staff I had to endure while being in the hospital.” | This category contains the general impression towards the staff. |
| 4.7.2 General Physicians | “…Excellent doctor…”; “The doctors and nursing staff are excellent…” | “There are many doctors that are not good use this hospital or work at this hospital.”; “I saw the most terrible doctor here…” | This category contains the general impression towards the physicians. |
| 4.7.3 General Nursing | “…The nurses are WONDERFUL... “; “…I was impressed with the nurses…” | “…the worst nursing staff in the world.”; “…PITIFUL STAFF THAT DOES NOT DESERVE TO BECOME NURSES!!!” | This category contains the general impression towards the nurses. |
| 4.7.4 General Administration |  | “…At first I thought that it was poor management…”; “…poor amin procedures in general…” | This category contains the general impression towards the hospital administration. |
| 4.7.5 General Others | “…The respiratory therapy staff was excellent…”; “…Kudos to the kitchen staff…” | “Heart echo team stink…”; “…Only complaint is their billing dept.” | This category contains the general impression towards other hospital employees. |
| 4.8 Availability | The following categories address the availability of the different hospital employees. | | |
| 4.8.1 Availability Staff/Overall | “…The day staff was better, or at least more visible…” | “…When I was there delivering my daughter no matter how many times I asked I couldn’t get anyone to take out the trash and had to use shopping bags to do it myself….” | This category describes if the staff is available to provide care. |
| 4.8.2 Availability Physicians | “…They have hospitalists now instead of the local doctor”; “…the hospitalists (sp) are exceptional doctors that are availibe in the hospital 24hrs…” | “…My doctor was not notified…”; “…The clinic portion has serious communication issues as it is very difficult (as far as I know, impossible) to actually talk to a doctor in person over the phone…” | This category describes if the physicians are available to provide care. |
| 4.8.3 Availability Nurses |  |  | This category describes if the nurses are available to provide care. |
| 4.8.4 Availability Administration |  |  | This category describes if the administration staff is available to take care of the patients. |
| 4.8.5 Availability Others |  | “…the A-team(as far as physicians)is definitely not around then.”; “…cleaning personnel=never see them…” | This category describes if other hospital employees are available to provide care. |
| 4.9 Communication | The following categories describe the communication flows between different hospital employees and the patients. | | |
| 4.9.1 Communication Staff/Overall | “…The staff is always friendly and informative. They did a great job of communicating expectations with my surgery; I knew exactly what to expect before, during, and after…”; “…The staff is very thoughtful and attentive. They do a great job explaining procedures…” | “Staff is very unfriendly, there is very little communication and questions go unanswered”; “…Had a nuclear stress test and to this day have never received an explanation of why my heart shut off…” | This category comprises how the staff communicates with the patients. |
| 4.9.2 Communication Physicians | “…Doctor was very busy but still had time to talk with me about what to do.”; “…Most of the MDs are polite and communicative…” | “Emergency room doctor was not listening to patient complaints failed to treat.”; “…Problem with communication between Doctors, Staff and Patients.” | This category comprises how physicians communicate with the patients. |
| 4.9.3 Communication Nursing | “…The nurses were all wonderful and explained what was going on to us every step of the way…”; “…The nurses were very cheerful and kept my spirits up and kept my family informed about me everyday.” | “Slower than average nursing staff with high concentration of English as second language making communication with impaired patients very difficult”; “Serveral nurses seemed like they did not want to answer my questions. Or wowuld try to find someone that would. communication poor…” | This category comprises how nurses communicate with the patients. |
| 4.9.4 Communication Administration |  |  | This category comprises how the administration staff communicates with the patients. |
| 4.9.5 Communication Others |  | “…The tech kept telling me they lost me. Never informed my partner…”; “The management is lacking in communication skills…” | This category comprises how other hospital employees communicate with the patients. |
| 5. Communication (Others) | The following categories describe administrative communication flows in the hospital. | | |
| 5.1 Scheduling |  | “…She wanted to scheduled an appointment first but was unavailable for 2 weeks.”; “…Hard to get an appointment” | This category characterizes the scheduling process or the process to get an appointment. |
| 5.2 Complaint Management | “…and the director taking immediate action to take care of him and then addressing our complaint…” | “…I've had bad times more than once and i have called to complain and make my voice known---it didn't change…”; “…When confronted them about their behavior they argued blaming a patient who had a heart attack was more important …” | This category contains how the hospital deals with complaints. |
| 6. Costs | The following categories address topics that belong to billing issues and costs. | | |
| 6.1 Amount of Costs | “…reasonable prices…” | “Overpriced medical services…”; “The Hospital bills are enormous…” | This category gives information how patients perceive the prices for hospital treatments. |
| 6.2 Only focused in money |  | “Their major interest was how much money they could run up on me and making sure i would pay it…”; “…The administration and hospital board care only about money and power…” | This category describes the attitude of the hospital if it only wants to make money with the care of patients. |
| 6.3 Insurance related issues |  | “…Our only issue was with billing, we kept getting conflicting stories on insurance quotes because we had two, they ended up using the non-primary insurance and primary and now we aren't even dealing with the hospital anymore…”; “…Since the doctor did not accept my insurance, I was charged the uninsured rate…” | This category focuses on issues related to the insurance of patients. For example, it informs about the acceptance of patients’ insurances and about the contracts of insurances with the hospital. |
| 6.4 Proper/Wrong billing |  | “…and I was charged for doctors that never even came into my room.”; “…They charged me for X-rays and services I didn't receive…” | This category characterizes about the accuracy of the billing. |
| 6.5 Early/Late billing | “…There is one thing they do good they bill you immediately!!” | “…WATCH OUT FOR THEIR BILL COLLECTORS AND EXPECT COLLECTION AGENCIES WITHIN 3 months…”; “…It took me 2 months to actually get one though (and it still was not clear)…” | This category contains information about the timing of the billing. |
| 7. Personal issues | The following categories are addressing issues due to personal feelings. | | |
| 7.1 Privacy | “…The unit was immaculate, quiet, and private…”; “…all people were very respectful as far as my wife's privacy…” | “made me lay on a gurney in the middle off a very busy hallway, in my gown, fully exposed while I waited for my ct scan. employees and patients kept walking by me…”; “…poor privacy…” | This category comprises personal issues regarding to the privacy of patients. |
| 7.2 Patient rights |  | “This hospital ignores you rights!...”; “…They have not been forthcoming with information regarding patients' and family's rights and responsibilities and seem extremely restrictive compared to other hospitals I have had family in - not allowing visits during normal daytime visting hours without valid explanation, for one example…” | This category comprises personal issues regarding to patient rights. |
| 8. Acknowledgements | The following categories are containing information about the gratitude of patients towards the different hospital employees. | | |
| 8.1 Acknowledgements Hospital | “…I recommend this hospital to all who ask and am thankful for it…”; “…Thank you Castle Medical Center.” | “…The practice and practice with your life. I will always hate this hospital and some day I will make it fair. Thank you !”; “…Thanks, for absolutely NOTHING!...” | This category describes if the patient is thankful towards the hospital. |
| 8.2 Acknowledgements Staff/Overall | “…thankful for it and it's compassionate staff.”; “…These doctors and their staff deserve to be acknowledged for the great care they give us vets. Thank you from one who is proud to be in such good hands.” | “…They bring me meds with no drink then look at me like I am stupid for not having one. THANKS…” | This category describes if the patient is thankful towards the staff. |
| 8.3 Acknowledgements Physicians | “…These doctors and their staff deserve to be acknowledged for the great care they give us vets. Thank you from one who is proud to be in such good hands.”; “…My thanks to Doctor Ellington…” |  | This category describes if the patient is thankful towards the physicians. |
| 8.4 Acknowledgments Nursing | “During my long stay at the ICU, the two nurses, Cheri and Duane were very cooperative and honest. They helped me all the way and were ready to help all the time. I wouldn't have to stay longer at this hospital if it wasn't for they trust and friendliness. Thank you.” | “…hours later the nurse came in to give me my morphine and then realized my pumps and meds had never been reconnected. THANKS…” | This category describes if the patient is thankful towards the nurses. |
| 8.5 Acknowledgements Others | “…Thanks again Jeff for helping my mom get out of the car and into a wheelchair.” |  | This category describes if the patient is thankful towards other employees. |
| 9. Recommendation | This category gives information about the patient satisfaction whether they recommend the hospital. | | |
|  | “…Would highly recommend to friends and family!”; “…I would highly recommend this hospital to anyone.” | “…I would NOT recommend this hospital Emergency Room to anyone I cared about.”; “…I would not recommend using this hospital.” | . |
| 10. Others | This category contains comments which could be assigned to any other categories. | | |
|  | “West Virginia Medical Card” | | This category contains comments which could be assigned to any other categories. |
